# Differential Contributions of the Complement Anaphylotoxin Receptors C5aR1 and C5aR2 to the Early Innate Immune Response against *Staphylococcus aureus* Infection

**DOI:** 10.3390/pathogens4040722

**Published:** 2015-10-23

**Authors:** Sarah A. Horst, Andreas Itzek, Andreas Klos, Andreas Beineke, Eva Medina

**Affiliations:** 1 Infection Immunology Research Group, Helmholtz Centre for Infection Research, Inhoffenstrasse 7, 38124 Braunschweig, Germany; E-Mail: sarah.horst@gmx.de; 2 Microbial Interactions and Processes Research Group, Helmholtz Centre for Infection Research, Inhoffenstrasse 7, 38124 Braunschweig, Germany; E-Mail: andreas.itzek@helmholtz-hzi.de; 3 Institute of Medical Microbiology and Hospital Epidemiology, Medical School Hannover, Hannover, Carl-Neuberg-Strasse 1, 30625 Hannover, Germany; E-Mail: Klos.Andreas@mh-hannover.de; 4 Institute for Pathology, University of Veterinary Medicine Hannover, Bünteweg 17, 30559 Hannover, Germany; E-Mail: andreas.beineke@tiho-hannover.de

**Keywords:** *Staphylococcus aureus*, C5a, C5aR1, C5aR2, complement system, neutrophils

## Abstract

The complement anaphylatoxin C5a contributes to host defense against *Staphylococcus aureus*. In this study, we investigated the functional role of the two known C5a receptors, C5aR1 and C5aR2, in the host response to *S. aureus.* We found that C5aR1^−/−^ mice exhibited greater susceptibility to *S. aureus* bloodstream infection than wild type and C5aR2^−/−^ mice, as demonstrated by the significantly higher bacterial loads in the kidneys and heart at 24 h of infection, and by the higher levels of inflammatory IL-6 in serum. Histological and immunohistochemistry investigation of infected kidneys at 24 h after bacterial inoculation revealed a discrete infiltration of neutrophils in wild type mice but already well-developed abscesses consisting of bacterial clusters surrounded by a large number of neutrophils in both C5aR1^−/−^ and C5aR2^−*/*−^ mice. Furthermore, blood neutrophils from C5aR1^−/−^ mice were less efficient than those from wild type or C5aR2^−/−^ mice at killing *S. aureus*. The requirement of C5aR1 for efficient killing of *S. aureus* was also demonstrated in human blood after disrupting C5a-C5aR1 signaling using specific inhibitors. These results demonstrated a role for C5aR1 in *S. aureus* clearance as well as a role for both C5aR1 and C5aR2 in the orchestration of the inflammatory response during infection.

## 1. Introduction

The complement system is an important component of the innate immune response and plays a crucial role in host defense against pathogens. Activation of the complement cascade leads to the formation of bioactive molecules such as C3a, C5a and C5b-9 that promote recruitment of immune cells to the site of infection and cell activation, the opsonophagocytosis of pathogens, and lysis of susceptible pathogens [1]. The relevance of the complement system in host defense against pathogens is emphasized by the increased susceptibility of patients with deficiency in the complement system to bacterial infections [2].

*Staphylococcus aureus* is an important pathogen as demonstrated by the severity of infections it can cause, its rising incidence, and increasing antibiotic resistance [3]. Like most other bacterial pathogens, invasion of the host by *S. aureus* generally results in the activation of the complement system. In a previous study, we have provided compelling evidence that complement activation and generation of anaphylatoxin C5a contributes to host defense against *S. aureus* bloodstream infection [4]. Thus, mice deficient in complement C5 were less able to control bacterial growth and exhibited higher levels of tissue damage in infected organs than C5-sufficient mice [4]. The protective role of C5 during *S. aureus* infections was demonstrated to be entirely mediated by the generation of C5a, since impairment of the membrane attack complex (MAC) formation, which is initiated by C5b, did not affect the resistance of mice to *S. aureus* infection [4]. The relevance of C5a for host defense against *S. aureus* is also exemplified by the multiple factors produced by this pathogen to interfere with this mediator or with its receptors. For example, the extracellular fibrinogen-binding protein (Efb) and its homologous extracellular complement-binding protein (Ecb) have been shown to block C5a generation and C5a-mediated neutrophil activation *in vitro,* as well as neutrophil recruitment into the peritoneal cavity in a mouse model of peritonitis [5]. Furthermore, staphylococcal superantigen-like 7 (SSL7) has been reported to inhibit C5a-mediated processes important for staphylococcal clearance [6].

C5a anaphylatoxin is a potent chemoattractant for phagocytic cells [7] and can stimulate oxidative burst in neutrophils thereby enhancing phagocytosis and bacterial killing in these cells [8]. It also has immunomodulatory properties such as regulation of cytokine expression in diverse cell types [9,10,11]. C5a exerts its biological functions after binding to the high-affinity receptors C5aR1 (CD88, C5aR) [12] and C5a-receptor-like-2 (C5aR2, C5L2) [13]. C5aR1 belongs to the family of G-protein-coupled receptors encompassing seven transmebrane segments and its binding to C5a results in calcium mobilization and triggering of several downstream signaling pathways [14,15]. In contrast, C5aR2 does not couple to G proteins and, partly for this reason, early studies proposed that C5aR2 is a nonsignaling decoy receptor competing with C5aR1 for C5a binding and thus preventing C5aR1 activation by removing C5a from the extracellular milieu [16,17,18]. Later studies however suggested an anti-inflammatory function for C5aR2 since it has been observed that blockades of C5aR2 with anti-C5aR2 antibody increased the levels of inflammatory IL-6 in septic rats [19] and genetic deletion of C5aR2 in mice enhanced the inflammatory responses to C5a [20]. Along the same lines, Bamberg and colleagues [21] showed that C5aR2 is predominantly intracellularly located, whereas C5aR1 is expressed on the plasma membrane. They also demonstrated that inhibition of C5aR2 by blocking antibodies did not alter the uptake or internalization of C5a [21]. These authors proposed that C5aR2 acts as an intracellular receptor that negatively modulates C5aR1-mediated response through the β-arrestin pathway [21]. On the other hand, evidence has been provided supporting a pro-inflammatory function of C5aR2 [22,23]. The functional role of C5aR2 in the inflammatory responses is at present highly controversial and may certainly have different roles in different systems and infection models [24].

Given the importance of C5a-dependent control of *S. aureus*, the objective of this study was to elucidate the role of C5aR1 and C5aR2 in host defense against *S. aureus.*

## 2. Results

### 2.1. Expression of C5aR1 and C5aR2 by Murine Neutrophils

**Figure 1:**
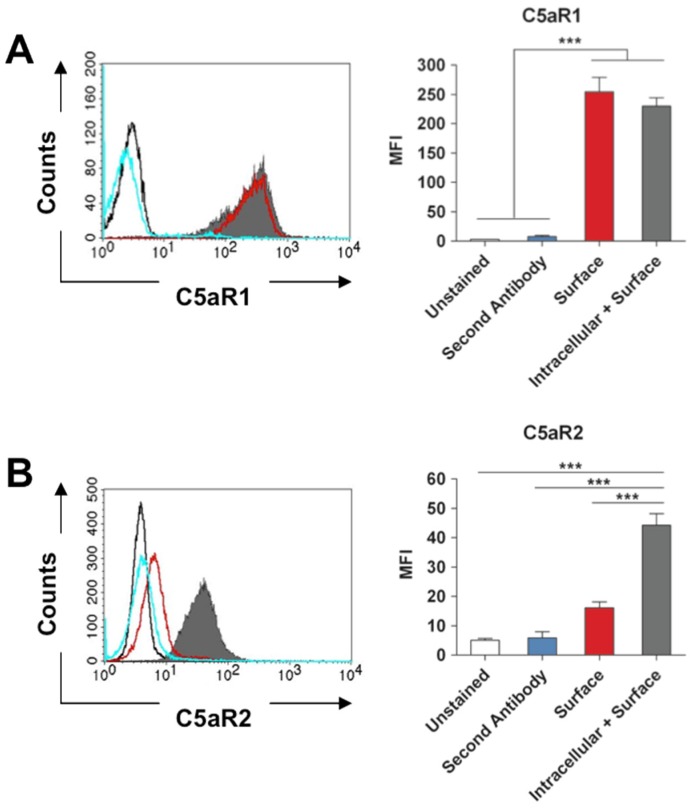
Expression patterns of C5aR1 and C5aR2 in murine neutrophils. (**A**) Histogram analysis (left) and the corresponding mean fluorescence intensity (MFI) quantification (right) of total C5aR1 expressed intracellularly/surface (solid grey histogram) or only on the surface (open red histogram) of murine neutrophils. (**B**) Histogram analysis (left) and the corresponding mean fluorescence intensity (MFI) quantification (right) of total C5aR2 expressed intracellularly/surface (solid grey histogram) or only on the surface (open red histogram) of murine neutrophils. In (**A**) and (**B**), open black histograms represent unstained control neutrophils and blue histograms represent neutrophils stained with irrelevant antibody (**A**) or with the secondary antibody alone (**B**). Histograms are representative of three independent experiments.

As we used a murine infection model to investigate the role of C5aR1 and C5aR2 in the host response to *S. aureus* in this study, we first determined the expression pattern of both anaphylatoxin receptors on murine neutrophils. For this purpose, neutrophils were isolated from murine bone marrow and the levels of intracellular and surface expression of C5aR1 and C5aR2 was determined by flow cytometry using specific antibodies. As shown in Figure 1A, C5aR1 was predominantly expressed on the surface of neutrophils. In contrast, most of C5aR2 molecules were intracellularly located in murine neutrophils with a very low level of C5aR2 detectable on the cell surface (Figure 1B). Thus, the pattern distribution of C5aR1 and C5aR2 in murine neutrophils is highly similar to that reported in human neutrophils [21].

### 2.2. C5aR1 But not C5aR2 Contributes to Bacterial Clearance in a Murine Model S. Aureus Bloodstream Infection

**Figure 2:**
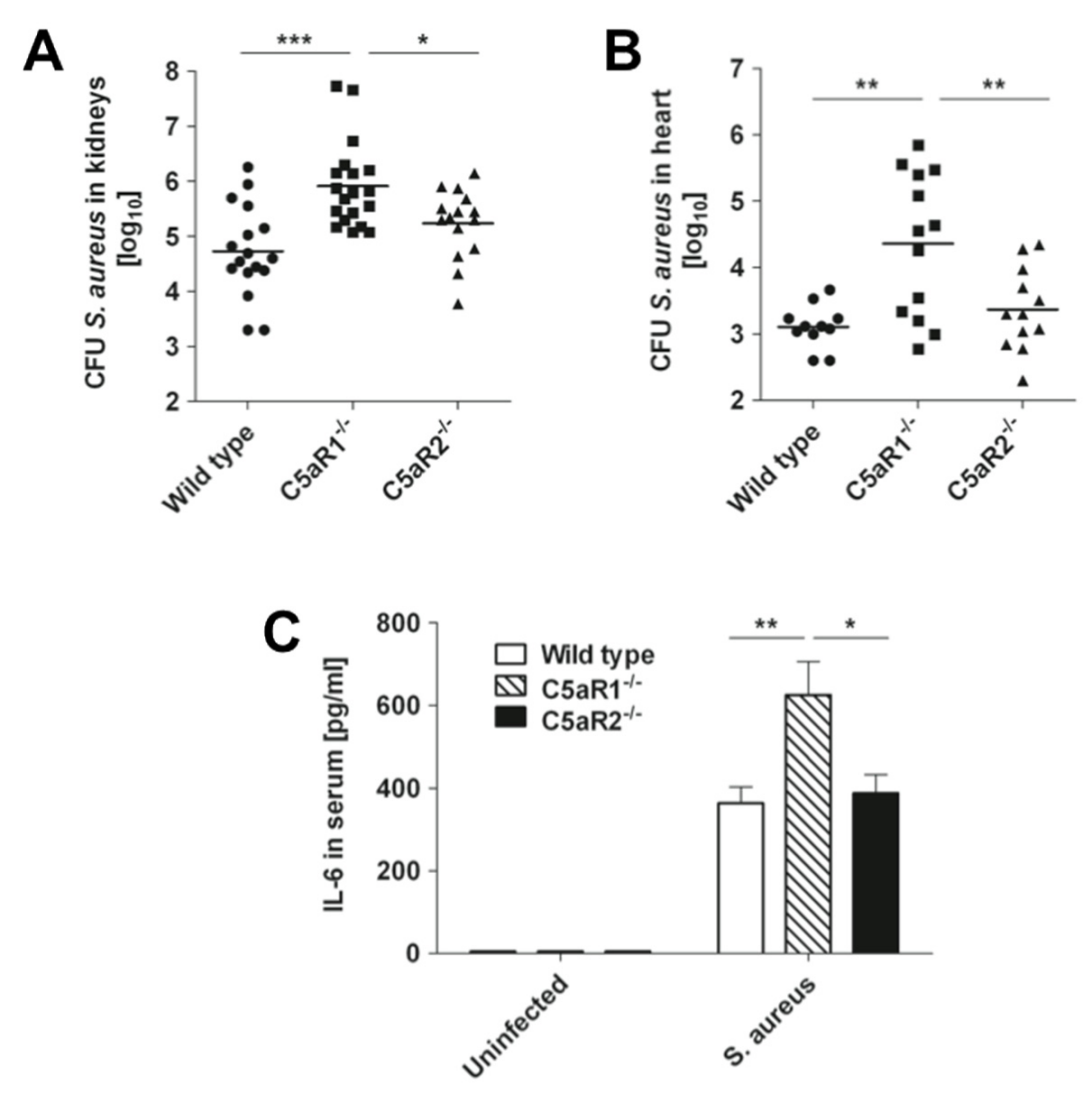
C5aR1 deficiency in mice is associated with greater bacterial loads. Bacterial loads in kidneys (**A**) and heart (**B**) of wild type (circles), C5aR1^−/−^ (squares), and C5aR2^−/−^ (triangles) mice at 24 h after intravenous infection with *S. aureus*. Each symbol represents one individual animal. Results from three independent experiments are shown. Horizontal lines indicate mean values. (**C**) Levels of IL-6 in serum of wild type (white bars), C5aR1^−/−^ (hatched bars) and C5aR2^−/−^ (black bars) mice at 24 h after intravenous infection with *S. aureus*. Each bar represents the mean ± SD of three independent experiments (n = 16). *, *p* < 0.05; **, *p* < 0.01; ***, *p* < 0.005.

As we previously showed that C5a has a protective role during *S. aureus* infection in mice, we investigated the specific C5a receptor mediating this protective effect. Wild type, C5aR1^−/−^, and C5aR2^−/−^ mice were intravenously infected with *S. aureus* and bacterial loads in kidneys and heart were determined at 24 h after bacterial inoculation. While the bacterial loads did not differ between C5aR2^−/−^ and wild type mice, C5aR1^−/−^ mice exhibited significant higher bacterial loads in kidneys (Figure 2A) and heart (Figure 2B) than wild type and C5aR2^−/−^ mice.

As high serum levels of pro-inflammatory cytokine IL-6 have been shown to contribute to severity of *S. aureus* infection [25], we determined the levels of serum IL-6 in wild type, C5aR1^−/−^, and C5aR2^−/−^ mice at 24 h after intravenous infection with *S. aureus*. The results in Figure 2C indicate that although the serum levels of IL-6 were increased in all *S. aureus-*infected groups, the IL-6 levels in serum of C5aR1^−/−^ were significantly higher than those in serum of wild type or C5aR2^−/−^ mice.

Together, these results indicate that C5a signaling through C5aR1 contribute to *S. aureus* clearance in this model of infection*.*

### 2.3. S. Aureus Abscess Formation is Accelerated in the Absence of C5aR1 or C5aR2

**Figure 3:**
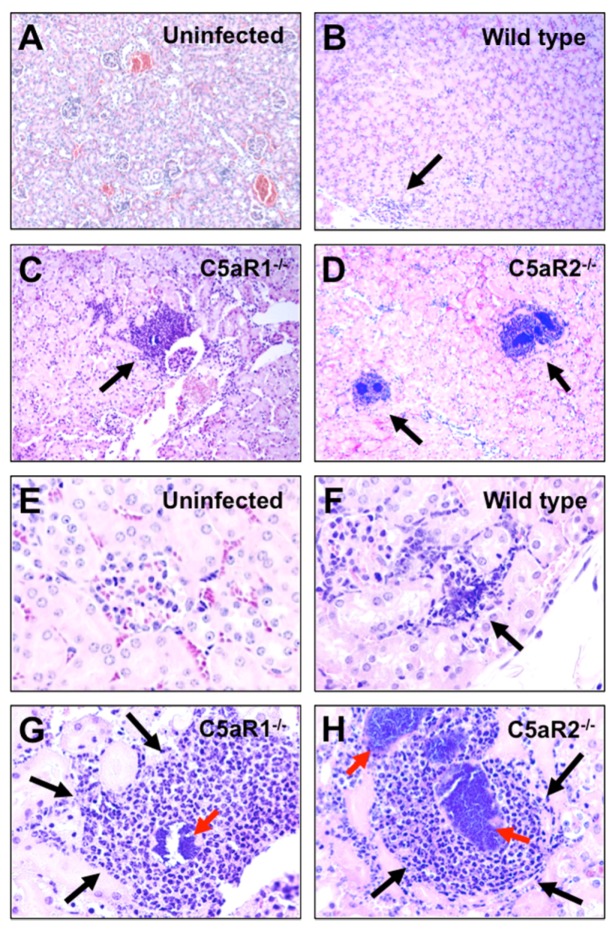
Accelerated abscess formation in the kidneys of *S. aureus-*infected C5aR1^−/−^ and C5aR2^−/−^ mice. H&E stained kidney sections of (**A**) and (**E**) uninfected control mice, (**B**) and (**F**) infected wild type, (**C**) and (**G**) infected C5aR1^−/−^, (**D**) and (**H**) infected C5aR2^−/−^ mice obtained at 24 h after intravenous *S. aureus* inoculation. Abscesses are indicated by black arrows. Bacterial clusters are indicated by red arrows. Magnification, ×10 in (**A**), (**B**), (**C**) and (**D**); ×40 in (**E**), (**F**), (**G**) and (**H**).

We next performed a histological examination of kidneys isolated from wild type, C5aR1^−/−^, and C5aR2^−/−^ mice at 24 h after *S. aureus* inoculation. While only small foci of inflammatory infiltrate cells with incipient abscess formation were observed in the kidneys of wild type mice (Figure 3B and F), multiple well-organized bigger abscesses exhibiting the typical central zone of a bacterial cluster surrounded by a cuff of inflammatory cells were visible in the kidneys of C5aR1^−/−^ (Figure 3C and G) and C5aR2^−/−^ (Figure 3D and H) mice already at this early time of infection.

**Figure 4:**
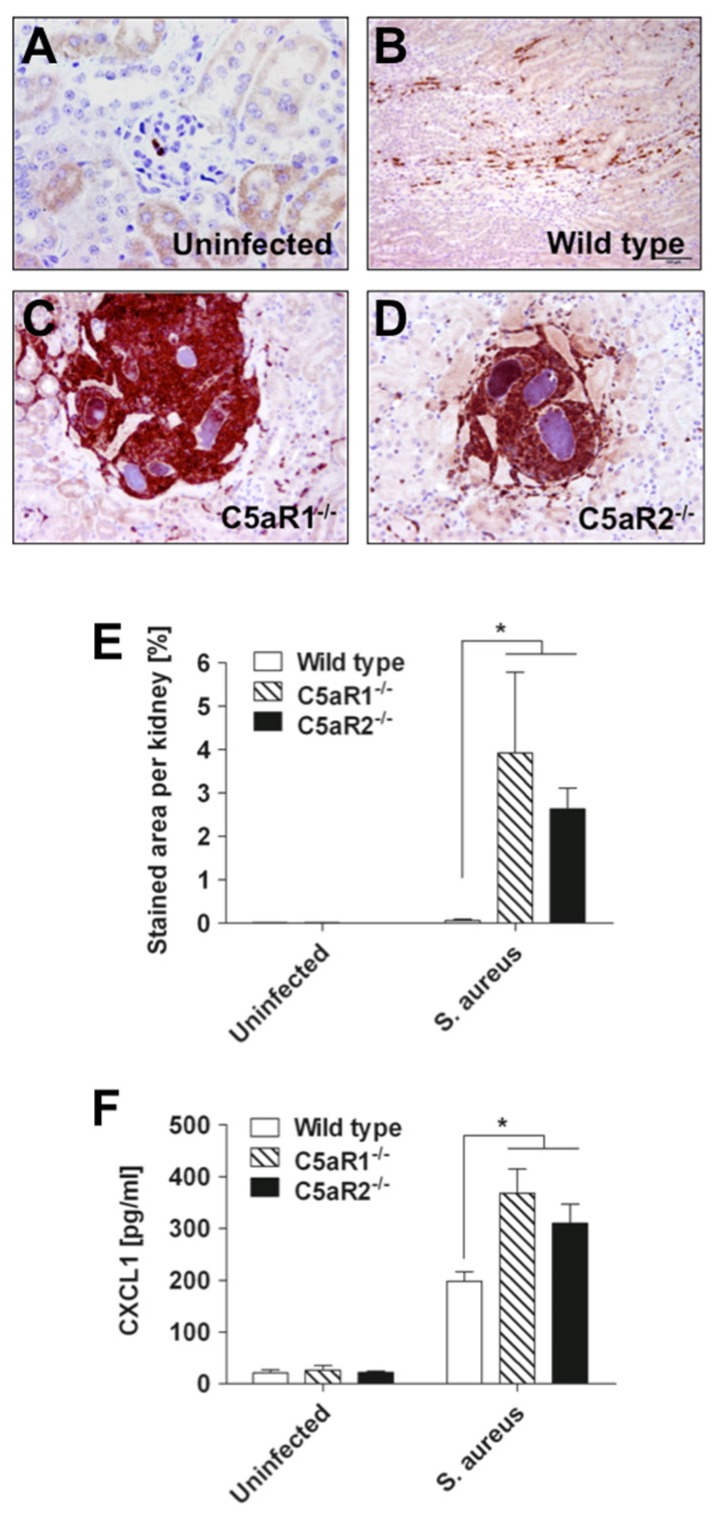
Increased infiltration of neutrophils in the kidneys of *S. aureus-*infected C5aR1^−/−^ and C5aR2^−/−^ mice. Kidney tissue obtained from (**A**) uninfected, (**B**) infected wild type, (**C**) infected C5aR1^−/−^ and (**D**) infected C5aR2^−/−^ mice at 24 h after intravenous *S. aureus* inoculation was stained with anti-Ly6G antibodies. Original magnification, ×40 in (**A**), (**C**) and (**D**); ×20 in (**B**). (**E)** Quantification of Ly6G immunostaining in kidney sections of uninfected or *S. aureus-*infected wild type (white bars), C5aR1^−/−^ (hatched bars) and C5aR2^−/−^ (black bars) mice at 24 h after bacterial inoculation. Ly6G-staining in kidneys was quantified by digitalizing the section with a color video camera mounted on an Axiophot microscope with a 10× objective. In total, four fields per section were evaluated. Data are presented as percentage of stained area per kidney. *, *p* < 0.05. (**F**) Concentration of CXCL1 in kidney homogenates of uninfected or *S. aureus-*infected wild type (white bars), C5aR1^−/−^ (hatched bars) and C5aR2^−/−^ (black bars) mice at 12 h after bacterial inoculation. Data are given as pg/mL of tissue homogenate and reported as mean ± SD. *, *p* < 0.05.

The larger abscesses in the kidneys of C5aR1^−/−^ and C5aR2^−/−^ mice induced higher levels of tissue destruction than the small emerging abscesses of wild type mice. Staining of kidney tissue with antibodies against Ly6G, a neutrophil surface marker, demonstrated that a large amount of inflammatory cells in the abscesses of C5aR1^−/−^ and C5aR2^−/−^ mice were neutrophils (Figure 4A–D). The quantification of neutrophils (Ly6G^+^ cells) infiltration into the kidneys of wild type, C5aR1^−/−^ and C5aR2^−/−^ mice is shown in Figure 4E. These observations were very interesting but a bit perplexing since one of the roles of C5a in the host response to pathogens is to contribute to the recruitment of neutrophils to the site of infection. An increased bacterial load cannot provide a satisfying explanation because that load was not increased in C5aR2^−/−^ mice as compared to wild type mice.

As CXCL1 is one of the major attractants of neutrophils in the mouse [26] and its production has been reported to be modulated by the C5a/C5aR1 signaling axis [27], we speculated that an increased production of this chemokine in the absence of either C5aR1 or C5aR2 might be responsible for the increased amount of neutrophils recruited to the kidneys of infected C5aR1^−/−^ and C5aR2^−/−^ mice. To test this hypothesis, we compared the levels of CXCL1 in kidneys homogenates of C5aR1^−/−^ and C5aR2^−/−^ mice with that of wild type mice at 12 h of infection. A significant increase in CXCL1 produced within the kidneys was demonstrated for all three groups of infected mice relative to uninfected controls (Figure 4F). However, comparison between mouse strains revealed that the levels of CXCL1 were significantly higher in the kidneys of C5aR1^−/−^ and C5aR2^−/−^ than in kidneys of wild type mice (Figure 4F). Thus, expression of CXCL1 preceded and positively correlated with the extent of neutrophil infiltration in the kidneys of the different groups of mice. These observations suggested that both C5a receptors might play an important role in the regulation of CXCL1 production in response to *S. aureus* and thereby in the coordination of the recruitment of neutrophils to the site of infection.

### 2.4. Signaling through C5aR1 is Required for Optimal Bactericidal Effect of Murine and Human Blood

**Figure 5:**
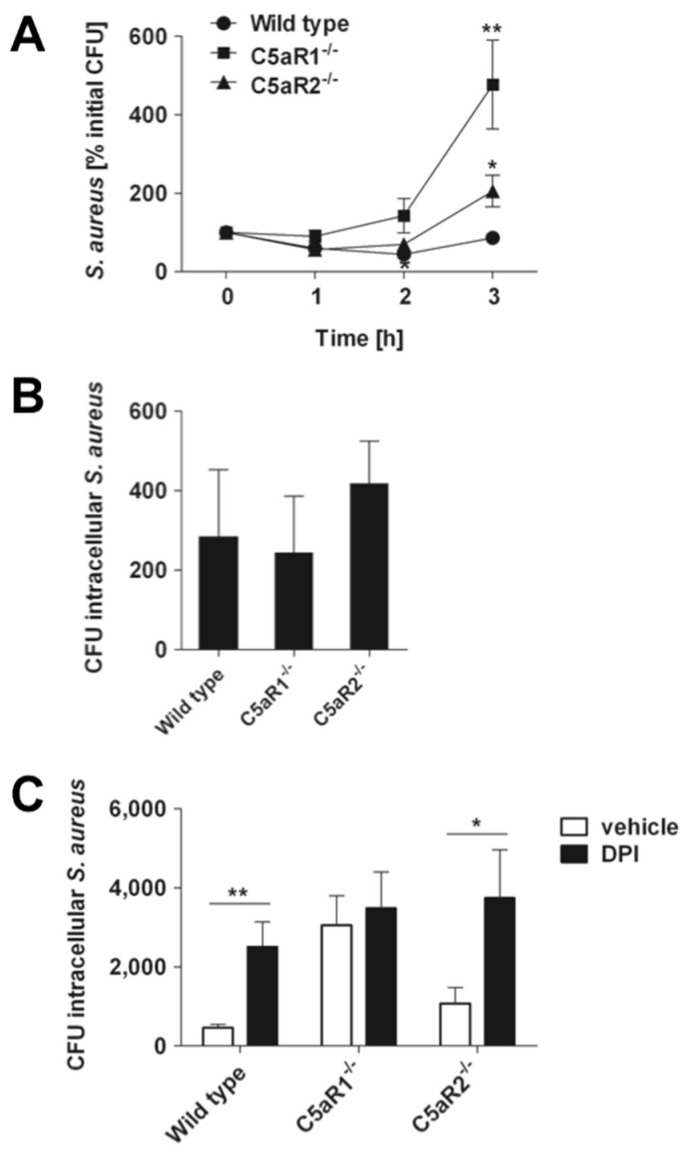
C5aR1 but not C5aR2 contributes to the bactericidal activity of murine blood neutrophils against *S. aureus*. (**A**) Kinetic of *S. aureus* growth in murine lepirudin-anti-coagulated whole blood drained from either wild type (circles), C5aR1^−/−^ (squares), or C5aR2^−/−^ (triangles) mice. Each symbol represents the mean ± SD of five independent experiments. *, *p* < 0.05 for C5aR1^−/−^ versus wild type; **, *p* < 0.01 for C5aR1^−/−^ versus C5aR2^−/−^. (**B**) Viable intracellular *S. aureus* at 0.5 h after bacterial inoculation of anti-coagulated murine whole blood. Data are expressed as the total number of bacteria per 10^6^ blood neutrophils. Bars represent mean ± SD from three independent experiments. (**C**) Viable intracellular *S. aureus* at 3 h after bacterial inoculation of anti-coagulated murine whole blood in the presence of the ROS inhibitor DPI (black bars) or incubated with vehicle alone (white bars). Data are expressed as the total number of bacteria per 10^6^ blood neutrophils. Bars represent mean ± SD from three independent experiments. *, *p* < 0.05; **, *p* < 0.005.

Since both C5aR1^−/−^ and C5aR2^−/−^ mice exhibited large numbers of neutrophils recruited to the infected kidneys but the amount of bacteria in this organ was significantly higher than in C5aR1^−/−^ mice, we hypothesized that neutrophils of C5aR1^−/−^ mice were less effective at killing *S. aureus* than those from C5aR2^−/−^ and wild type mice. To test this hypothesis, we compared the bactericidal capacity of blood neutrophils from C5aR1^−/−^ with that of C5aR2^−/−^ and wild type mice using a lepirudin anti-coagulated whole blood assay. The results in Figure 5A show that while *S. aureus* growth was controlled in blood samples from wild type and C5aR2^−/−^ mice, the bactericidal capacity of blood from C5aR1^−/−^ mice against *S. aureus* was impaired and resulted in progressive bacterial growth.

To confirm that the bactericidal activity in the whole blood assay was mediated by neutrophils, blood cells were harvested by centrifugation at 0.5 and 3 h after *S. aureus* inoculation, treated with 5 µg/mL lysostaphin to kill extracellular bacteria, and the amount of viable intracellular staphylococci was determined after lysing the neutrophils. The amount of intracellular viable bacteria did not differ between wild type, C5aR1^−/−^ and C5aR2^−/−^ mice at 0.5 h after bacteria inoculation, indicating that neither C5aR1 nor C5aR2 influenced the phagocytic uptake of *S. aureus* by neutrophils (Figure 5B). However, the amount of viable intracellular *S. aureus* bacteria was significantly higher in blood neutrophils from C5aR1^−/−^ mice than in those from wild type or C5aR2^−/−^ mice (Figure 5C).

These results indicate that deletion of C5aR1 impaired the intracellular killing of *S. aureus* in neutrophils. The superior bactericidal effect of neutrophils from wild type and C5aR2^−/−^ mice was mediated by ROS since addition of DPI, an inhibitor of ROS production by NADPH oxidase (nicotinamide adenine dinucleotide phosphate-oxidase), significantly decreased the capacity of wild type and C5aR2^−/−^ to kill *S. aureus* but it didn’t affect the amount of intracellular viable bacteria in neutrophils of C5aR1^−/−^ mice (Figure 5C). The results imply that C5aR1 signaling enhanced the intracellular killing of *S. aureus* in neutrophils and that this process is dependent on ROS production.

**Figure 6:**
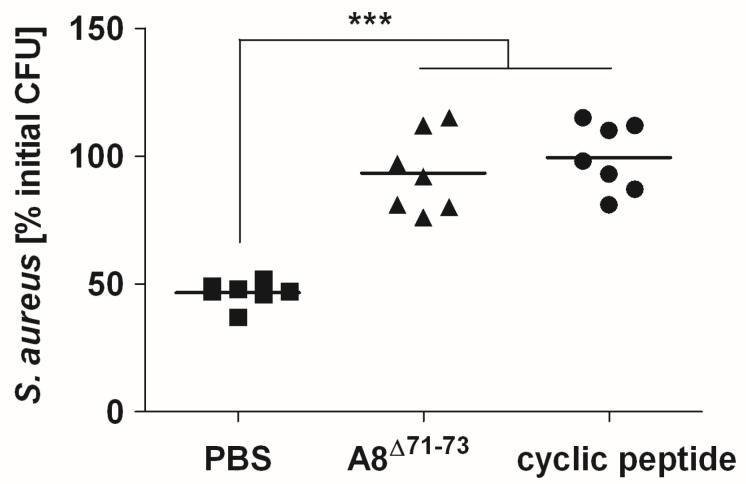
C5aR1 but not C5aR2 contributes to bactericidal activity of human blood against *S. aureus*. Viable *S. aureus* after 2 h incubation in lepirudin-anti-coagulated human blood in the presence of PBS (squares), the C5aR1 antagonists A8^Δ71–73^ (triangles), or cyclic peptide (circles). Each symbol represents one donor. Cumulative data from three independent experiments is shown. Horizontal lines represent mean values. ***, *p* < 0.005.

We validated the results obtained in the murine model in the human system by evaluating the effect of agents capable of blocking specifically C5aR1 (cyclic peptide) or both C5aR1 and C5aR2 (A8^Δ71–73^) on the bactericidal capacity of human blood against *S. aureus*. Like the murine experimental system, the bactericidal effect of human blood against *S. aureus* was significantly impaired after blockage of C5aR1 alone or both C5aR1 and C5aR2 (Figure 6).

## 3. Discussion

The complement component C5a plays an important role in host defense against *S. aureus* infection [4]. In the present study, we investigated the role of the C5a receptors C5aR1 and C5aR2 in mediating the protective effect of C5a in a murine model of *S. aureus* bloodstream infection. Our results provide clear evidence for a role of C5aR1 but not of C5aR2 in bacterial clearance. Thus, while C5aR1^−/−^ mice exhibited higher bacterial loads in systemic organs than wild type or C5aR2^−/−^ mice, the bacterial loads in the organs of wild type and C5aR2^−/−^ mice were comparable. Using a whole blood assay as well as isolated blood neutrophils, we demonstrated that phagocytosis of *S. aureus* was preserved in C5aR1^−/−^ neutrophils but the capacity to kill internalized *S. aureus* bacteria was highly impaired in comparison to that of neutrophils of wild type and C5aR2^−/−^ mice. These results also indicated that C5aR1 signaling boosted the bactericidal capacity of neutrophils as reported in other experimental settings [28]. By using specific inhibitors capable of blocking C5a-C5aR1 signaling, we could also show that C5aR1 was required for the killing of *S. aureus* in human blood. Deletion of C5aR2 did not affect bacterial clearance in infected mice, or the capacity of blood neutrophils to eliminate intracellular *S. aureus*.

More interesting was the observation that genetic deletion of either C5aR1 or C5aR2 resulted in enhanced production of neutrophil chemoattractant CXCL1 and in the infiltration of neutrophils into the infected kidneys, accelerated abscess formation and increased tissue damage. These results were rather unexpected since one of the functions of C5a is to promote the influx of neutrophils to the site of infection [29] and therefore we envisioned that disruption of C5a signaling after deletion of its receptor would impair neutrophil recruitment to the site of infection. A potential explanation for this inconsistency is that C5a receptors need to cooperate with other innate immune recognition receptors to mount an appropriate immune response during *S. aureus* infection. For example, it has been reported that C5a enhanced secretion of TLR4-induced inflammatory cytokines such as IL-6 and TNF-alpha by human monocytes, and in human macrophages through the crosstalk with other TLRs, such as TLR3 and TLR7 [30]. Therefore, it seems that the outcome of the C5a receptor/TLR crosstalk strongly depends on the cell type, on the specific TLR involved in pathogen recognition and on the particular C5a receptor engaged. The integration of signaling pathways triggered by the different receptors may be required for tailoring the immune response to the specific pathogen and for maintaining the fine balance between protective immunity and inflammatory pathology. Disruption of this network by deletion of C5a receptors could result in the shifting of the immune response from bacterial clearance to exacerbate inflammation and associated tissue damage as observed in our study. Due to the relevance of these immune networks, many pathogens have developed strategies to subvert host immunity and promote their survival through manipulation of the crosstalk interactions between the different immune receptors [31]. This is well exemplified by the diverse mechanisms evolved by *S. aureus* to highjack the C5a receptors and dysregulate the innate immune response for its own benefit. Thus, the Panton-Valentine leukocidin PVL and the γ-hemolysin HlgC/HlgB bind to the human C5aR1 and C5aR2 and, in particular, the binding of the PVL component, LukS-PV to C5aR1 induces potent inhibition of C5a-induced immune cell activation [32,33,34]. Several strains of *S. aureus* can also release an exoprotein called CHIPS for chemotaxis inhibitory protein (CHIPS), which acts as a potent and specific inhibitor of neutrophil and monocyte chemotaxis toward anaphylatoxin C5a and formylated peptides [35,36]. CHIPS has been reported to exert its inhibitory functions by binding to C5aR1 [37]. An alternative explanation could be that both C5a receptors cooperated somehow during *S. aureus* infection as suggested by others in different settings [21,22] and blockage of C5aR1 might enhance the effect of an isolated C5aR.

In summary, the results of our study demonstrated that both C5aR1 and C5aR2 are required for an appropriate host response to *S. aureus* bloodstream infection. While only C5aR1 is involved in bacterial clearance, both C5aR1 and C5aR2 seem to be important for the orchestration of the inflammatory response during infection.

## 4. Experimental Section

### 4.1. Bacteria

The *S. aureus* strain SH1000 used in this study expresses γ-hemolysin, a virulent determinant capable of binding C5aR1 and C5aR2 [33], and low levels of exoproteins in a similar way to that observed for many clinical isolates [38,39]. *S. aureus* was grown to Mid-Log phase in brain heart infusion medium (BHI, Roth, Karlsruhe, Germany) at 37 °C with shaking (120 rpm), collected by centrifugation, washed with sterile PBS, and diluted to the required concentration. The number of viable bacteria was determined after serial diluting and plating on BHI-agar.

### 4.2. Mice and Infection

Specific pathogen-free, 8–12 weeks-old C57BL/6 female mice were purchased from Harlan Winkelmann (Borchen, Germany). C5aR1^−/−^ mice (C57BL/6-C5aR11(tm1-Cge)) [40] and C5aR2^−/−^ (C57BL/6-Gpr77(tm1-Cge)) [20] mice were bred in house under pathogen free conditions. All animals were provided with food and water ad libitum, and housed in groups of up to 5 mice per cage in individually ventilated cages. Mice were infected with 1 × 10^7^ CFU of *S. aureus* in 100 µL of PBS via a tail vein and sacrificed at 24 h after bacterial inoculation by CO_2_ asphyxiation. The bacterial load was enumerated in kidney and heart homogenates in PBS plating 10-fold serial dilutions on blood agar plates.

Animal experiments were performed in strict accordance with the German regulations of the Society for Laboratory Animal Science (GV-SOLAS) and the European Health Law of the Federation of Laboratory Animal Science Associations (FELASA). The animal experiments included in this study comply with the ‘3R’. Thus, the minimum amount of mice absolutely necessary for running statistical analysis was included in each experiment (Reduction). Mice were monitored in daily basis for weight loss and sign of pain or distress. However, these parameters were marginal during the short time of infection selected for this study (24 h) (Refinement). When possible, infections were performed *in vitro* using murine blood (Replacement). All experiments were approved by the ethical board Niedersächsisches Landesamt für Verbraucherschutz und Lebensmittelsicherheit, Oldenburg in Germany (LAVES; permit N. 33.9-42502-04-10/0296).

### 4.3. Staining of C5aR1 and C5aR2

Myeloid cells were obtained by flushing the bone marrow from tibiae and femurs of healthy mice. Neutrophils were isolated using the “Neutrophil Isolation Kit” according to the manufacturer’s instruction (Miltenyi Biotec, Bergisch Gladbach, Germany). C5aR1 and C5aR2 expression was determined using rabbit PE-conjugated anti-mouse C5aR1 (Biolegend, San Diego, USA), or rabbit anti-mouse C5aR2 (Hycult Biotechnology, Uden, The Netherlands) combined with FITC-conjugated anti-rabbit-IgG (Sigma, St. Louis, USA) as secondary antibody. Neutrophils were incubated for 30 min at 4°C in the dark in PBS/2% FCS and analyzed using a FACSCalibur flow cytometer (BD Biosciences, Heidelberg, Germany). For intracellular staining, neutrophils were fixed with 4% paraformaldehyde and permeabilized with 0.5% saponin in PBS/2% FCS.

### 4.4. Cytokine Determination

Levels of IL-6 in the serum and of CXCL1 in kidneys homogenates of *S. aureus-*infected mice were determined by ELISA according to the manufacturer’s recommendations (BD Biosciences).

### 4.5. Histology and Immunohistochemistry

Mice were euthanized and whole kidneys were excised, fixed in 10% formalin, embedded in paraffin, sectioned at a thickness of 5 µm and stained with hematoxylin and eosin (H&E) for histological examination.

Immunohistochemistry was performed using a monoclonal rat anti-mouse Ly6G-specific antibody (clone 1A8; BioLegend), which is a surface marker expressed predominantly by neutrophils. For blocking of the endogenous peroxidase, formalin-fixed, paraffin-embedded tissue sections were treated with 0.5% H_2_O_2_ diluted in methanol for 30 min at room temperature. Subsequently, sections were heated in 10 mM Na-citrate buffer pH 6.0 for 20 min in a microwave oven (800 W). Following blocking with 20% goat serum for 30 min, sections were incubated with the primary antibody (dilution 1:500) for 1.5 h at room temperature followed by incubation with the secondary antibody (biotinylated rabbit-anti-rat immunoglobulin; BA 4001; Vector Laboratories, Peterborough, UK) also for 30 min at room temperature. Slides were subsequently incubated with the peroxidase-conjugated avidin-biotin complex (PK 6100; Vector Laboratories) for 30 min. After visualization of the positive antigen-antibody reaction by incubation with 3.3-diaminobenzidine-tetrachloride (DAB) for 5 min, sections were counterstained with hematoxylin and evaluated by light microscopy.

Ly6G-staining in kidneys was quantified by digital image analysis of stained kidney sections using a color video camera (Color View II, 3.3 Megapixel CCD; Soft Imaging System, Münster, Germany) mounted on an Axiophot microscope (Zeiss, Oberkochen, Germany) with a 10× objective. In total, four fields per section from three independent samples were evaluated. The immunolabeled areas were measured using the analysis 3.1 software package (Soft Imaging System) and expressed as percentage of stained area per kidney [41,42].

### 4.6. Bactericidal Assay in Murine Blood

A total of 10^4^ *S. aureus* bacteria were added to 1 ml of murine blood treated with the anti-coagulant lepirudin (50 µg/mL), which does not interfere with complement activation [8]. Blood was incubated at 37°C under rolling conditions (6 rpm) in borosilicate glass tubes with silicone rubber seal. Bacterial killing was assessed by plating aliquots on agar plates at 1, 2 and 3 h after inoculation. Percentage of bacterial survival was calculated referred to the starting inoculum.

For intracellular killing, blood cells were harvested by centrifugation for 5 min at 500 *× g* 0.5 and 3 h after inoculation with *S. aureus*. The pellet was treated with 5 µg/mL lysostaphin for 5-10 min at room temperature to kill extracellular bacteria, washed twice with PBS and cells were lysed using 0.1% Triton-X in dH_2_O. Bacterial counts were enumerated by plating serial dilutions on blood agar plates and results were expressed as number of intracellular bacteria per 10^6^ neutrophils. The number of neutrophils in blood samples was determined using a VetScan HM5 Hematology System (Abaxis).

In some experiments, ROS production was inhibited by adding 10 µg/mL of Diphenyleneiodonium (DPI) to blood samples 30 min prior to bacterial inoculation.

### 4.7. Bactericidal assay in Human Blood

A total of 2 × 10^6^ bacteria were added to 1 ml of lepirudin-anticoagulated (50 µg/mL) whole human blood obtained from healthy volunteers after obtaining signed informed consent and incubated for 2h at 37°C under rolling conditions in glass tubes in the presence or absence of 6.4 µM C5aR1 antagonists A8^Δ71–73^, a gift from Jörg Köhl (University of Lübeck, Lübeck, Germany), or 5 µM cyclic peptide AcF-[OPdChaWR], provided by Werner Tegge (HZI, Braunschweig, Germany). The optimal concentrations of these inhibitors were determined in preliminary experiments. The number of viable bacteria was determined in serially diluted blood on blood agar. Bacterial survival was expressed as percentage of the original number of inoculated bacteria.

### 4.8. Statistical analysis

Data were analyzed using GraphPad Prism 5. Results are presented as mean ± SD. Comparison between groups was performed by one-way ANOVA and Tukey’s post hoc test. *P* values < 0.05 were considered as significant.
